# The telomerase activator TA-65 protects from cigarette smoke-induced small airway remodeling in mice through extra-telomeric effects

**DOI:** 10.1038/s41598-022-25993-7

**Published:** 2023-01-16

**Authors:** Arnaud Jean Florent Tiendrébéogo, Thibaud Soumagne, François Pellegrin, Maylis Dagouassat, Jeanne Tran Van Nhieu, Philippe Caramelle, Emmanuel N. Paul, Benjamin Even, Maeva Zysman, Yvon Julé, Abdoulaye Samb, Jorge Boczkowski, Sophie Lanone, Frédéric Schlemmer

**Affiliations:** 1 IMRB, INSERM U955, 94000 Créteil, France; 2 Université Paris Est-Créteil, Faculté de Santé, 94000 Créteil, France; 3 Laboratoire de physiologie et d’explorations fonctionnelles physiologiques, Université Cheik Anta Diop, Dakar, Senegal; 4 Assistance Publique Hôpitaux de Paris, Hôpitaux Universitaires Henri Mondor, Département de Pathologie, 94000 Créteil, France; 5 BioCellvia, Marseille, 13006 France; 6 Assistance Publique Hôpitaux de Paris, Hôpitaux Universitaires Henri Mondor, Service d’explorations fonctionnelles respiratoires, DHU A-TVB, FHU Senec, 94000 Créteil, France; 7 Assistance Publique Hôpitaux de Paris, Hôpitaux Universitaires Henri Mondor, Unité de Pneumologie, DHU A-TVB, FHU Senec, 94000 Créteil, France

**Keywords:** Chronic obstructive pulmonary disease, Preclinical research, Target identification

## Abstract

Small airway remodeling (SAR) is a key phenomenon of airflow obstruction in smokers, leading to chronic obstructive pulmonary disease (COPD). SAR results in an increased thickness of small airway walls, with a combination of peribronchiolar fibrosis with increased fibrous tissue and accumulation of mesenchymal and epithelial cells. SAR pathogenesis is still unclear but recent data suggest that alterations in telomerase activity could represent a possible underlying mechanism of SAR. Our study was dedicated to identify a potential protective role of TA-65, a pharmacological telomerase activator, in a cigarette smoke (CS) model of SAR in mice, and to further precise if extra-telomeric effects of telomerase, involving oxidative stress modulation, could explain it. C57BL/6J mice were daily exposed to air or CS during 4 weeks with or without a concomitant administration of TA-65 starting 7 days before CS exposure. Morphological analyses were performed, and mucus production, myofibroblast differentiation, collagen deposition, as well as transforming growth factor-β1 (TGF-β1) expression in the small airway walls were examined. In addition, the effects of TA-65 treatment on TGF-β expression, fibroblast-to-myofibroblast differentiation, reactive oxygen species (ROS) production and catalase expression and activity were evaluated in primary cultures of pulmonary fibroblasts and/or mouse embryonic fibroblasts in vitro. Exposure to CS during 4 weeks induced SAR in mice, characterized by small airway walls thickening and peribronchiolar fibrosis (increased deposition of collagen, expression of α-SMA in small airway walls), without mucus overproduction. Treatment of mice with TA-65 protected them from CS-induced SAR. This effect was associated with the prevention of CS-induced TGF-β expression in vivo, the blockade of TGF-β-induced myofibroblast differentiation, and the reduction of TGF-β-induced ROS production that correlates with an increase of catalase expression and activity. Our findings demonstrate that telomerase is a critical player of SAR, probably through extra-telomeric anti-oxidant effects, and therefore provide new insights in the understanding and treatment of COPD pathogenesis.

## Introduction

Chronic obstructive pulmonary disease (COPD) is the most frequent chronic respiratory disease and remains a major public health problem. It is one of the main causes of mortality, and its morbidity, although more difficult to appreciate, is undeniable. Cigarette smoking remains the major risk factor to develop COPD in industrialized countries, although other factors may represent important causes in developing countries and/or in the context of specific working activities^1,2^. COPD is characterized by a progressive decline in lung function with an irreversible airflow limitation. It is classically associated with two distinct anatomic lesions, small airway remodeling (SAR) and emphysema, which may both participate in the pathophysiology of this obstructive lung disease. Emphysema is defined by a loss of alveolar walls and a subsequent enlargement of alveolar spaces, while SAR is occurring in small airways (internal diameter of less than 2 mm in humans) without cartilage and results in an increased thickness of airway walls^3–5^. SAR involves subepithelial, smooth muscle and peribronchiolar compartments of the airway wall. It is characterized by a variable combination of peribronchiolar fibrosis, with increased fibrous tissue, and accumulation of mesenchymal and inflammatory cells in the walls of the membranous and respiratory bronchioles. It is now recognized that SAR occurs early in the course of the disease and its contribution to airflow limitation in COPD exceeds that of the decreased elastic recoil due to emphysema^5,6^.

Relatively few studies address the pathogenesis of SAR in COPD, as compared to those related to the pathogenesis of emphysema. According to these studies, it appears that cytokines, chemokines and/or receptors such as Interleukin-1β, growth factors, Tumor Necrosis Factor Receptor I and II, as well as proteases/antiproteases and oxidants/antioxidants imbalance play a role in cigarette smoke (CS)-induced SAR^7–12^. Recent studies also suggest that premature aging could represent a possible mechanism underlying SAR pathogenesis^13–15^. As they may counteract the progressive shortening of telomeres characterizing premature aging, pharmacological activators of telomerase activity are considered as an interesting approach for the treatment of premature aging-related diseases, including COPD^14,16^. However, if considering SAR as an early process in COPD pathogenesis, the potential benefits of telomerase activators on SAR might preferentially be due to their ability in enhancing some of the extra-telomeric functions of this enzymatic complex. Indeed, non-canonical functions of telomerase, that are non-obligatory extra-telomeric, may influence various essential cellular processes like gene expression, signaling pathways, mitochondrial function, cell survival and stress resistance^17^, some of them probably remaining unknown.

TA-65, a small molecular compound isolated from the traditional Chinese medicine plant *Astragalus membranaceus* in the early 2000's^18^, was initially identified in an empirical screening based on its ability to upregulate basal telomerase activity levels in neonatal human keratinocytes^19^. It has been shown to improve markers of metabolic, bone, and cardiovascular health in the general population^20,21^, and to improve the macular function in patients suffering from early age-related macular degeneration^22^. The potential effect of TA-65 in COPD, in particular on SAR, has never been studied so far. Therefore, the current study was dedicated to investigate a protective role of TA-65 against CS-associated SAR genesis, by using an experimental model of SAR based on the daily exposure of young mice to CS during 4 weeks. Because a telomeric effect of telomerase activation was unlikely to explain a potential benefit from TA-65 treatment in our model, and as telomerase deficiency had been described as promoting oxidative stress by reducing catalase activity^23^, we further address if a modulation of the cellular oxidative stress level through an increase of catalase activity could participate in the protective effect of TA-65 treatment.

## Methods

### Statement

The study is reported in accordance with ARRIVE guidelines. All methods were performed in accordance with the relevant guidelines and regulations.

### Mice

Male C57BL/6 mice (8 weeks old) were purchased from Janvier (Le Genest-St-Isle, France) and acclimated during one week. All mice were supplied with food (SAFE, Auguy, France) and tap sterilized water ad libitum in standard wire-topped cages in a controlled environment, with a 12 h light/dark cycle.

### Experimental model of SAR

Mice were randomly divided into eight experimental groups (7–8 mice in each group) according to the treatment they received: air or cigarette smoke [CS(−) and CS(+) animals respectively] ± TA-65 or a telomerase inhibitor, Imetelstat, or their respective vehicle (see below). Exposure to CS was performed daily for 2 h, during 28 consecutive days, as previously described^24^. Briefly, smoke was generated from 3R4F scientific cigarettes (University of Kentucky, Louisville, Kentucky) and introduced into the exposure chamber using a 60 mL syringe. CS(−) mice were daily exposed to air during the same amount of time and under the same contention conditions. One hour prior to air or CS exposure, mice were treated with TA-65 (TA-Sciences, Geron Corporation, USA) or Imetelstat (GRN163L, gracious gift from Geron^®^). The telomerase activator TA-65 (25 mg/kg body weight in DMSO, final concentration 6.25 mg/mL) or vehicle (DMSO) was administrated to animals by oral gavage. The telomerase inhibitor, Imetelstat (45 mg/kg in saline) or vehicle (saline) was injected intraperitoneally to animals. These pharmacological treatments began one week before air or CS exposure, and were not associated with any complication.

### Lung tissue samples

One day after the last exposure to air or CS, mice were sacrificed by intraperitoneal injection of an anesthesia cocktail (xylazine plus ketamine diluted in physiological serum) followed by exsanguination (abdominal aortic section). Lungs were removed, inflated in situ at a constant pressure of 25 cm H_2_O with 10% buffered formalin for 20 min before paraffin embedding and further histological and immunohistochemical analysis.

### Histological and immunohistochemical analysis

Paraffin-embedded lung tissue sections (5 μm) were stained with hematoxylin–eosin (HE) for histological examination. The occurrence, localization and severity of histological lesions were assessed using a semi-quantitative score adapted from Roggli^25^. At least 10 fields per lung tissue section were analyzed (magnification 20×), and a scale of four semi-quantitative scores was used (0: normal; 1: +; 2: ++; 3: +++) to estimate broncho-vascular edema, inflammation and the presence of scalloped bronchi. To measure small airway thickness, the lung sections were scanned at 20× magnification using a NanoZoomer-SQ and digital images of lung sections were captured using the NDP.view 2 software (Hamamatsu Corporation, Hamamatsu, Japan). The selection of small bronchi was based on their Feret diameter (100–300 µm) using a dedicated software (ImageJ 1.44H, NIH, USA). Images of randomly selected bronchi were extracted from the digital images of the entire lung sections and deleted manually from their surrounding parenchymal tissue and vessels using Gimp 2.8 software (Free Software Foundation Inc.). Software image analysis was then performed on these cleaned digital images (ImageJ 1.44H, NIH, USA). Bronchial wall thickness was measured at five different equidistant points along the bronchial wall allowing the estimation of the mean bronchial thickness. As a confirmation method, a dedicated software program (Biocellvia, France) has been especially developed and allowed automatical delineating and measurement of the bronchial wall thickness of selected bronchi, with a pixel size of 0.454 µm allowing both a high-resolution visualization of morphological structure and an accurate measurement of bronchi morphometric parameters. The wall thickness was assessed from thousands midpoints distributed in the whole length of the wall bronchi and expressed as a mean ± standard error of the mean (SEM).

To go further into the characterization of small airway thickness, lung sections were stained with Picro-Sirius Red for total collagen deposition or Periodic Acid Schiff (PAS) for mucus production, respectively. Moreover, tissue sections were immunostained with the following antibodies: mouse anti-α-SMA (1:3000, Sigma A5228, France), rabbit anti-collagen I (1:100, Abcam ab21286, France), rabbit anti-collagen III (1:200, Abcam ab7778), rabbit anti-vimentin (1:200, Abcam ab92547), mouse anti-TGF-β1 (1:100, Abcam ab64715), rabbit anti-TERT (1:500, Abcam ab104588). Negative controls were assessed using goat anti-rabbit (Vector, BA-1000) or anti-mouse IgG (BA-9200) instead of primary antibodies. After incubation with primary antibodies overnight at 4 °C or 2 h at room temperature, slides were washed with phosphate-buffered saline tween 0.05% (Sigma, USA), incubated with an appropriate biotinylated secondary antibody (Vector Laboratories, USA). Diaminobenzidine or alkaline phosphatase (Vector Laboratories, USA) solutions were used as immunoreaction revelation substrates. Sections were counterstained with hematoxylin.

To quantify the immunostainings, slides were coded and masked for identity to perform a double-blinded analysis. Pictures of randomly selected microscopic fields (magnification, 20×) were taken using identical conditions for light setting and contrast thanks to an optical microscope AXIOPLAN 2 (Carl Zeiss AG, Germany) coupled to a digital camera (Carl Zeiss AG, Germany). Only small airways, i.e. with an internal diameter ranging from 100 to 300 µm, were considered for analysis and at least 10 bronchioles that met these criteria were analyzed on each slide. Color segmentation analyses in the Image J software was used to quantify immunostainings.

### Mouse primary lung fibroblast (MPLF) cultures

#### Isolation of fibroblasts

Primary cultures of lung bronchial fibroblasts were established from nine to twelve weeks-old male C57Bl/6 mice as previously described^26^. Briefly, medium size airways were dissected free of surrounding tissue and opened longitudinally. Lung tissue explants were diced into fine pieces (around 1 mm^3^) and cultured in complete medium: Dulbecco’s modified Eagle’s medium (DMEM, Life Technologies, Cergy-Pontoise, France) supplemented with 12.5% fetal bovine serum (GE healthcare, UK) and 1% antibiotic solution (Life Technologies). After three washing in complete medium at 37 °C, tissue explants were placed into 24-wells tissue culture plate with 400 µL of complete medium per well. Tissue explants were then cultured at 37 °C in 5% CO_2_, with fresh medium added every other day. After 14 days, tissue explants were removed and cells were cultured one more week until they reached 90% confluence; at that time, cells were harvested using trypsin/EDTA solution (Life Technologies). Cell suspensions were seeded in 25 cm^3^ flasks and referred to as a passage 1 culture. At passage 3, isolated cells were characterized as fibroblasts by morphological appearance and expression pattern of specific proteins by immunocytochemistry: positive immunostaining for vimentin (fibroblasts marker—1:100; Dako, Denmark) associated with negative immunostaining for cytokeratin (bronchial epithelial cells marker—1:50; Dako, Denmark), and CD31 (endothelial cells marker—1:50; Dako). Each culture contained between 8 to 10% of α-SMA positive cells (1:200, Sigma A5228).

#### Mouse embryonic fibroblasts (MEF) cell line

MEF were cultured in DMEM supplemented with 12.5% fetal calf serum, 1 mM sodium pyruvate, 10 mM HEPES, 10 U/mL penicillin and 10 µg/mL streptomycin.

### Fibroblast-to-myofibroblast differentiation assay

To assess the effect of TA-65 on fibroblast-to-myofibroblast differentiation, MPLF or MEF cells seeded on Lab-Tek chamber slides were treated with recombinant TGF-ß1 (10 ng/mL, Sigma T7039, France) for 96 h in presence or absence of TA-65 (2 μM) or Imetelstat (1 μM). After a first wash, cells were fixed with 4% paraformaldehyde at 4 °C for 10 min, then permeabilized with 0.1% Triton X-100 during 15 min at room temperature, washed and blocked with 2% bovine serum albumin in Dulbecco’s phosphate-buffered saline at room temperature for 30 min. Slides were subsequently incubated with monoclonal mouse anti-α-SMA (1:200, Sigma A5228) in a humidified chamber overnight at 4 °C. The fluorescent signal was detected using a goat anti-mouse secondary antibody (1:500, conjugated with Alexa 488, Life technologies). Slides were mounted using ProLong DAPI (Life Technologies). A fluorescence microscope coupled to a digital camera utilizing the Axiovision^®^ software was used to view and acquire images (Zeiss, Jena, Germany). The quantification of signal was performed by using Image J software.

### Telomerase activity

Telomerase activity was quantified with a TeloTAGGG Telomerase PCR ELISA^PLUS^ kit as per the manufacturer instructions (Roche Diagnostics, Meylan, France).

### Oxidative stress quantification and catalase expression and activity

Intracellular reactive oxygen species **(**ROS) production was quantified by measuring H2-DCFH-DA (2,7-dichlorofluorescein diacetate) oxidation using a specific probe (Life Technologies, France). Catalase expression levels were evaluated by immunostaining (1:300; Abcam, France) as previously described^27^. Catalase activity was quantified with an Amplex Red Catalase Assay kit (Life Technologies, France). Probes and kits were used following the manufacturer’s protocols.

### Statistical analysis

Differences among experimental groups were examined by one-way analysis of variance (Kruskal–Wallis test) followed by Dunn’s post hoc test. Values for all data were expressed as mean ± S.E.M. A p value less than 0.05 was considered statistically significant. Analyses were performed using Prism software (GraphPad 6.0).

### Ethics

All experiments were approved by our local Institutional Animal Care and Use Committee (Comité d'éthique—ComEth, ANSES/ENVA/UPEC, #11/12/12-3).

## Results

### Cigarette smoke exposure induced small airway remodeling in mice

**Figure 1:**
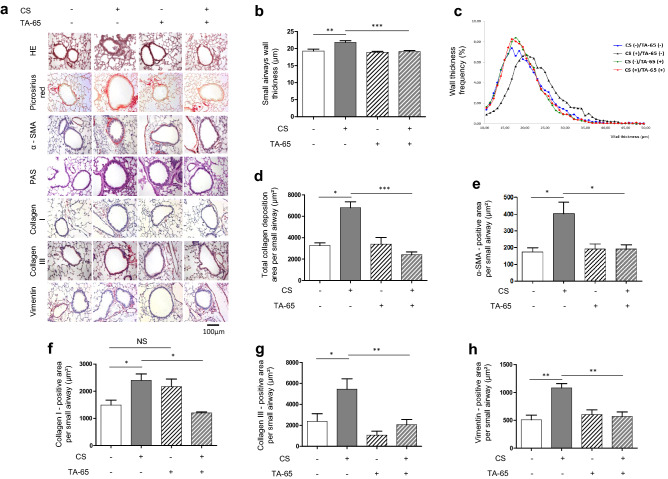
TA-65 effects on cigarette smoke-induced small airway remodeling. Representative lung tissue sections of mice exposed to air or cigarette-smoke (CS) with or without TA-65, and stained for HE, picrosirius red, α-SMA, PAS, Collagen I, Collagen III, or Vimentin (**a**). Quantification of small airways wall thickness by manual [(**b**), in mean] or automated method [(**c**), distribution], α-SMA expression (**d**), PAS staining (**e**), Collagen I (**f**), Collagen III (**g**) and Vimentin (**h**) expression. Original magnification, ×20. n = 7–8 mice per group. Data are presented as mean ± SEM. *p < 0.05, **p < 0.01, ***p < 0.001.

Figure 1 shows representative images of lung tissue sections obtained after exposure of mice, during 4 consecutive weeks, to room air [CS(−)] or cigarette smoke [CS(+)] (Fig. 1a). Histological analysis showed no difference between the different groups for broncho-vascular edema, inflammation and the presence of scalloped bronchi (data not shown). Morphometric analysis of the small airways stained with HE showed a significant increase of the small airway wall thickness in CS-exposed mice as compared to air-exposed mice, by using two different methods for the assessment of bronchial wall thickness (Fig. 1b,c). Moreover, the quantification of total collagen deposition (Fig. 1d), α-SMA (Fig. 1e), collagen I (Fig. 1f), collagen III (Fig. 1g) and vimentin (Fig. 1h) positive area per small airway revealed a significant increase of those fibrosis markers in CS-exposed mice as compared to air-exposed animals. As attended, no positive staining for PAS could be detected in small airways, although it was present in the large airways of CS-exposed mice (Fig. 1a and data not shown). Overall, these results demonstrate that exposure of mice to CS during 4 weeks leads to the development of small airway remodeling characterized by an increase of small airway wall thickness and peri-bronchiolar fibrosis.

### TA-65 protects from CS-induced small airway remodeling

**Figure 2:**
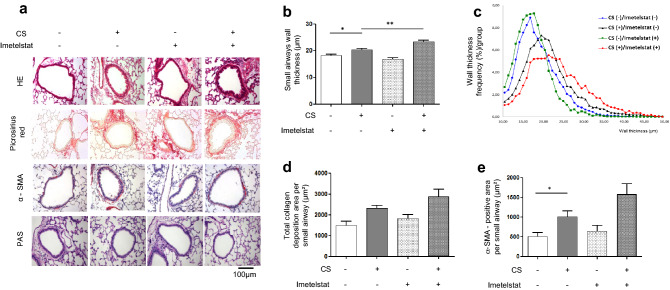
Imetelstat effects on cigarette smoke-induced small airway remodeling. Representative lung tissue sections of mice exposed to air or cigarette-smoke (CS) with or without Imetelstat, and stained for HE, picrosirius red, α-SMA, or PAS (**a**). Quantification of small airways wall thickness [mean in (**b**), distribution in (**c**)], α-SMA expression (**d**), PAS staining (**e**). Original magnification, ×20. n = 7–8 mice per group. Data are presented as mean ± SEM. *p < 0.05, **p < 0.01.

To address whether telomerase activation could protect from CS-induced small airway remodeling, mice were treated with TA-65, a known activator of telomerase activity^18^. As shown in Supplementary Fig. S1, this treatment led to the increased expression of the telomerase TERT subunit in all TA-65(+) animals in epithelial as well as mesenchymal cells. This was accompanied by a protection against CS-induced SAR: small airway wall thickness, α-SMA, total collagen deposition, collagen I and III and vimentin-positive area per small airway were similar to those of unexposed animals (CS(−)/TA-65(−)) (Fig. 1). None of these parameters was modified after TA-65 treatment alone (CS(−)/TA-65(+) versus CS(−)/TA-65(−) experimental groups). Mice treatment with the inhibitor of telomerase activity Imetelstat confirmed the implication of telomerase in CS-induced small airway thickening, as CS(+)/Imetelstat(+) mice showed increased small airway walls thickening as compared to CS-only exposed animals (Fig. 2a–c). A similar increase, although non-significant, was observed for α-SMA and total collagen deposition in CS(+)/Imetelstat(+) mice (Fig. 2a,d,e).

### TA-65 protects from CS-induced TGF-β1 overexpression

**Figure 3:**
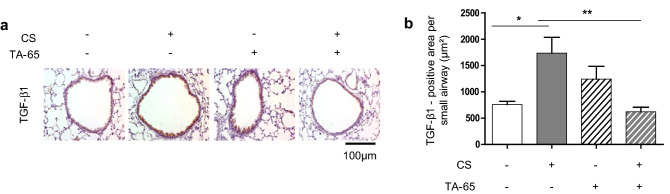
TGF-β1 expression. Representative lung tissue sections of mice exposed to air or cigarette-smoke (CS) ± TA-65, and subjected to TGF-β immunostaining (**a**). Quantification of TGF-β expression (**b**). Original magnification, ×20. n = 7–8 mice per group. Data are presented as mean ± SEM. *p < 0.05, **p < 0.01.

Transforming growth factor-β (TGF-β) is extensively involved in the development of lung fibrosis^28^. We therefore addressed the issue whether the small airway fibrosis developed after CS exposure was accompanied by TGF-β1 overexpression, and whether this phenomenon could be modulated by TA-65 treatment. As expected, we observed that CS exposure led to an increased expression of TGF-β1, which was mainly detectable in bronchial epithelial cells (Fig. 3). TA-65 treatment protected from CS-induced TGF-β1 overexpression: TGF-β expression in CS(+)/TA-65(+) mice was similar to that of CS(−), whatever the TA-65 status. No significant modification of TGF-β expression was observed after TA-65 treatment alone (CS(−)/TA-65(+) versus CS(−)/TA-65(−)).

### TA-65 protects from TGF-β1-induced myofibroblast differentiation in primary culture of lung fibroblasts

**Figure 4:**
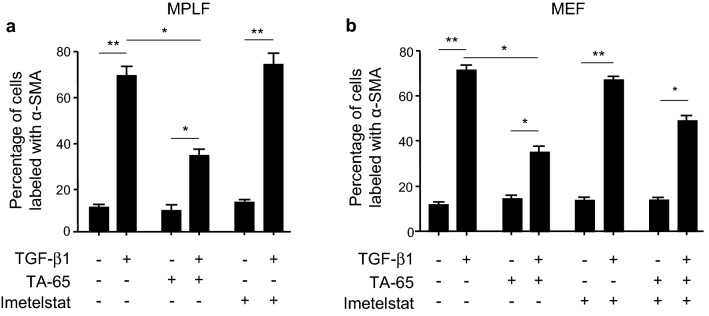
Effects of TA-65 on TGF-β-induced fibroblasts-to-myofibobroblasts differentiation in vitro. Mouse primary lung fibroblasts (MPLF) and MEF cells were pre-treated with TA-65 (2 µM) or Imetelstat (1 µM) during 24 h, followed by TGF-β treatment (10 ng/mL) for 48 h. Histograms show quantification of α-SMA expression evaluated by immunostaining (**a**: MPLF; **b**: MEF). Representative images of cells stained with α-SMA monoclonal antibody are available in Supplementary Fig. S3. n = 3 samples per group. Data are presented as mean ± SEM. *p < 0.05, **p < 0.01.

The origin of myofibroblast in fibrotic diseases in general and SAR in particular could be multifactorial. However, a previous study excludes pericytes and two epithelial cell populations as the origin of myofibroblasts in lung fibrosis, giving the fibroblast a central role for myofibroblast origin^29^. We therefore focused on fibroblasts as the final common effector of fibrogenesis pathway to assess in vitro whether the modulation of telomerase activity could interfere with fibroblast-to-myofibroblast differentiation. Thus, we postulated that TGF-β1 produced by bronchial epithelial cells in response to cigarette smoke exposure would act on mesenchymal cells in a paracrine manner. We first confirmed that TA-65 increases telomerase activity in vitro, both in mouse primary lung fibroblasts (MPLF) and mouse embryogenic fibroblasts (MEF) (Figure S2). We then observed that the increase in α-SMA expression described in fibroblasts after exposure to TGF-β1 was abolished when cells were treated with TA-65 in vitro (Fig. 4 and Supplementary Fig. S3). In accordance with our in vivo results, no modification of α-SMA expression was observed in cells treated with TGF-β1 in combination with Imetelstat as compared to TGF-ß1 treated cells.

### TA-65 decreases TGF-β1-induced ROS production and modulates catalase in murine fibroblasts

**Figure 5:**
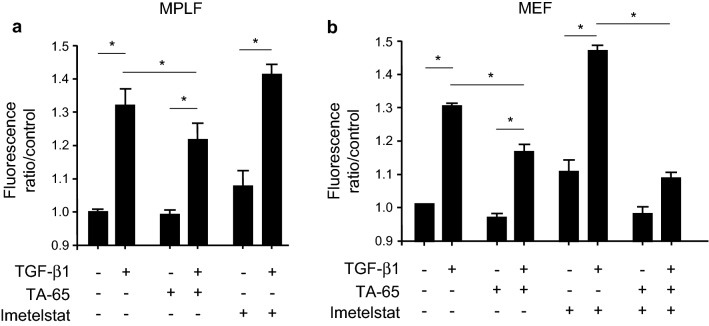
Effects of TA-65 on TGF-β1-induced ROS production in vitro. Mouse primary lung fibroblasts (MPLF) and MEF cells were pre-treated with TA-65 (2 µM) or Imetelstat (1 µM) during 24 h, followed by TGF-β treatment (10 ng/mL) for 48 h. Quantification of intracellular reactive oxygen species **(**ROS) production by measuring H2-DCFH-DA (2,7-dichlorofluorescein diacetate) oxidation in MPLF (**a**) and MEF (**b**). n = 3–4 samples per group. Data are presented as mean ± SEM. *p < 0.05.

To specify if the modulation of myofibroblast differentiation by TA-65 could correlate with a variation in cellular oxidative stress level, we evaluated TGF-β1-induced ROS production in MPLF and MEF cells. As shown in Fig. 5a, exposure of MPLF to TGF-β1 induced a significant increase in total ROS production. This increase was significantly less if the fibroblasts were treated with TA-65, whereas Imetelstat had no significant effect on TGF-β1-induced ROS production. Similar results were observed with MEF cells (Fig. 5b), in which the co-treatment with TA-65 and Imetelstat led to the same result as with TA-65 treatment alone.

**Figure 6:**
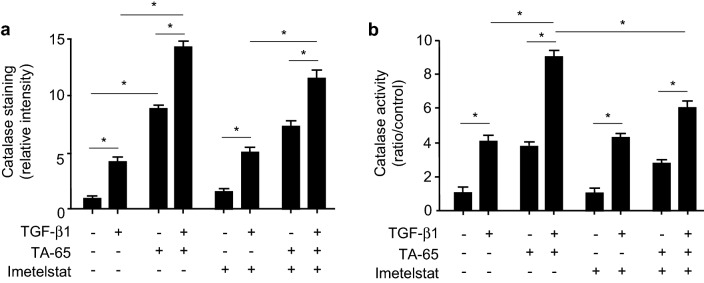
Effects of TA-65, Imetelstat and TGF-β1 on catalase in vitro. MEF cells were pre-treated with TA-65 (2 µM) and/or Imetelstat (1 µM) during 24 h, followed by TGF-β treatment (10 ng/mL) for 48 h. Catalase expression was evaluated by immunostaining (**a**). Representative images are available in Supplementary Fig. S4. Catalase activity of cell lysates was quantified using an Amplex Red Catalase Assay kit (**b**). n = 4 samples per group. Data are presented as mean ± SEM. *p < 0.05.

In order to determine the mechanisms involved in the modulation of oxidative stress induced by TA-65 treatment, we evaluated the expression and activity of one of the main anti-oxidant enzymes, catalase, in MEF cells. When treating MEF cells with TA-65, we observed an increase in both catalase expression (Fig. 6a and Supplementary Fig. S4) and activity (Fig. 6b). Moreover, catalase expression and activity further increased when cells were treated concomitantly with TGF-β1. While Imetelstat alone had no obvious effect on catalase expression and activity as compared to controls (treated or not with TGF-β1), it attenuated TA-65 effects on catalase activity when cells were treated concomitantly with both drugs.

## Discussion

Overall, our data demonstrate that cigarette smoke (CS)-induced SAR is prevented by TA-65, a pharmacological activator of telomerase. This protective effect is associated with a decreased expression of the pro-fibrotic cytokine TGF-β1 in the small airway walls of CS-exposed mice, together with a protection against fibroblast-to-myofibroblast differentiation in response to TGF-β1 in lung primary fibroblast in vitro. Postulating TGF-β1 expressed by bronchial epithelial cells after CS exposure may play a significant role in peribronchial fibrosis genesis, our study provides the first evidence of the potential implication of telomerase in CS-induced SAR.

In mice, the different features of COPD can be modelled by exogenous administration of proteases, chemicals, particulates, or CS^30^. However, given that CS remains the predominant cause of COPD in patients, animal models using exposure to CS appear as the most relevant to the human disease. The large majority of the literature on CS-exposed mice has been dedicated to the study of emphysema, probably because of the initial interest for this anatomic lesion following the seminal study by Gross and colleagues in the early 60’s^31^. Despite it is now well accepted that SAR constitutes the earliest and predominant determinant of airflow obstruction in smokers and COPD patients^5,6,32^, small airways have been poorly investigated so far in animal models of COPD^33–35^. Interestingly, we were able to induce SAR as early as after 4 weeks of CS exposure, whereas previous studies have shown similar bronchial lesions but after exposure of mice, rats or guinea pigs during at least 8 weeks^34–42^. Thus, our murine model of CS-induced SAR offers great opportunities to identify new therapeutic targets for the early phases of COPD. Indeed, studying animals early after the initiation of CS exposure (i.e. before the establishment of emphysema or detectable airflow limitation) can provide clues to some pathogenic mechanism(s) at a stage where the disease may have a greater possibility of reversibility^42^.

The regulation of telomerase activity is multifactorial in mammalian cells, involving telomerase gene expression, post-translational protein–protein interactions, as well as protein phosphorylation^43^. We chose here to modulate telomerase activity using TA-65, a pharmacological telomerase activator known to upregulate telomerase activity via a MAPK-dependent pathway^18,44^. Evidence from the literature shows that the effects of telomerase activity modulation are highly dependent on the cell type, particularly during fibrotic processes^45,46^. Therefore, we addressed the issue of telomerase activity modulation in a single cellular population, i.e. on pulmonary fibroblasts, taken as the final effectors of fibrosis pathogenesis. Moreover, as TERT expression is correlated with telomerase activity in many cell types, the results we obtained in mice lung tissue sections stained for TERT corroborate the efficiency of TA-65 administration in vivo. Finally, as treatment of mice with the telomerase inhibitor, Imetelstat, aggravates SAR in response to CS, one can speculate that TA-65 effects involves the activation of telomerase.

We gave TA-65 treatment over 4 weeks and, as others did before, reasoned that any given effect of this treatment would reflect telomerase activity independently of its effects on telomere length^47^. This is also supported by the fact that mice have extremely long telomeres (10 times that of humans), that makes it difficult to associate the observed effects with telomere length variation^48,49^. The modulation of at least two major cell processes could account for the protective effect of TA-65 on fibroblast-to-myofibroblast differentiation that we observed both in vivo and in vitro: TGF-β signaling pathway and epithelial-mesenchymal transition (EMT), the implication of the latter in our in vivo model being not formally excluded. Indeed, Yang et al*.* have demonstrated that a drug essentially composed of *Astragalus membranaceus* extracts was able to exert anti-fibrotic effects by interacting with TGF-β/Smad signaling in myofibroblasts^50^. Furthermore, in a mouse model of liver fibrosis as well as TGF-β-exposed myofibroblasts, administration of this compound inhibits the phosphorylation of two essential downstream mediators of TGF-β pathway, Smad2 and Smad3, together with decreasing the expression of α-SMA as well as JNK phosphorylation^50^. This is in accordance with our results in vitro showing that TA-65 treatment protects from fibroblast-to-myofibroblast differentiation in response to TGF-β1. Moreover, it has been recently shown that *Astragalus membranaceus* ameliorates renal interstitial fibrosis by inhibiting tubular EMT both in vivo and in vitro, via the inhibition of TGF-β1 expression^51^. Finally, telomerase-deficient mice have been shown to have high levels of TGF-β1^47^, which could be linked to the results we obtained in mice, showing a decreased TGF-β1 expression in animals treated with TA-65. Altogether, and given the respective role of TGF-β signaling and EMT in fibrosis pathogenesis, these events could be responsible for TA-65 protective effects in our CS-induced SAR model.

We finally aimed to investigate if the beneficial effects of TA-65 (and Imetelstat as conversely detrimental) we observed could involve a modulation of TGF-β1-induced cellular oxidative stress, as previously suggested^23^. We observed that TA-65 induced a significant decrease of ROS production in cells exposed to TGF-β1 in vitro, which could be related to an increase in catalase expression and activity, thus enhancing a feed-back loop in which TGF-β1-induced ROS production stimulates the catalase-dependent anti-oxidant system. However, as we did not observe a significant effect of Imetelstat on ROS production and catalase expression and activity, it remains uncertain that the effects of TA-65 (or Imetelstat) we observed in vivo are the direct consequences of catalase (and/or ROS levels) modulation by TA-65 (or Imetelstat) we observed in vitro. In addition, although we did not observed significant changes in NADPH oxidase 1, 2 and 4 expression, Glutathione peroxidase-1 expression and activity, as well as any significant changes in mitochondrial ROS production in our system (data not shown), we cannot exclude that other pro- or anti-oxidants pathways might also be involved^52,53^. Furthermore, we did not addressed if TA-65 effects on fibroblast-to-myofibroblast differentiation and upregulation of ROS are synergistic or unrelated outputs of TGF-β1 in our system. It is well established that TGF-β1 and ROS both play a role in CS-induced SAR pathophysiology^11,40^. Their role in the induction of fibroblast-to-myofibroblast differentiation has been previously reported, although it was not specifically assessed in SAR models^54^. In a bleomycin-induced lung fibrosis model, Sato et al. demonstrated, using NOX4 knockdown and *N*-acetylcysteine treatment, that NOX4-derived ROS generation was critical for TGF-β-induced Smad phosphorylation and myofibroblast differentiation^55^. In addition, Gorowiec et al*.* showed that hydrogen peroxide (H_2_O_2_) was able to induce EMT and to promote the expression of TGF-β1, whereas the inhibition of TGF-β1 signaling as well as treatment with antioxidants were able to prevent the oxidative stress driven EMT-like changes^56^. Finally, Bocchino et al. investigated the impact of oxidative stress in the establishment of the IPF fibroblasts phenotype and showed that this IPF phenotype was inducible upon oxidative stress (H_2_O_2_ exposure) in control cells and was sensitive to ROS scavenging^57^. Taking into account the complex interplay between ROS and TGF-β1, we assume they might have synergistic effects on myofibroblast differentiation in our experimental model, but this remains to be confirmed.

Nevertheless, the clinical relevance of our results can be attested by a recent study demonstrating that mutations of telomerase represent a risk factor for COPD susceptibility, at a frequency that is similar to alpha-1 antitrypsin deficiency, a long-known Mendelian cause for emphysema^58,59^.

## Conclusions

We demonstrate for the first time a protective effect of telomerase activation, using TA-65, in CS-induced SAR in mice. This work provides new insights for the development of novel therapeutic approaches specifically dedicated to the early stage of COPD.

## Supplementary Information

Supplementary Figure S1.

Supplementary Figure S2.

Supplementary Figure S3.

Supplementary Figure S4.

Supplementary Legends.

## Data Availability

The dataset supporting the conclusions of this article is included within the article and its additional files.

## Abbreviations

α-SMA: α-Smooth muscle actin
COPD: Chronic obstructive pulmonary disease
CS: Cigarette smoke
DNA: Deoxyribonucleic acid
EMT: Epithelial–mesenchymal transition
HE: Hematoxylin–eosin
JNK: Jun N-terminal kinases
MAPK: Mitogen-activated protein kinases
MEF: Mouse embryogenic fibroblasts
MPLF: Mouse primary lung fibroblasts
NIH: National institutes of health
PAS: Periodic acid Schiff
ROS: Reactive oxygen species
SAR: Small airway remodeling
SEM: Standard error of the mean
TERT: Telomerase reverse transcriptase
TGF-β: Transforming growth factor-β
TRAP: Telomerase repeats amplification protocol
