# Supplementary figures and images for: The telomerase activator TA-65 protects from cigarette smoke-induced small airway remodeling in mice through extra-telomeric effects
Source: Sci Rep. 2023 Jan 16;13:25. doi: 10.1038/s41598-022-25993-7 (PMC9842758; doi:10.1038/s41598-022-25993-7)

## Slide 1
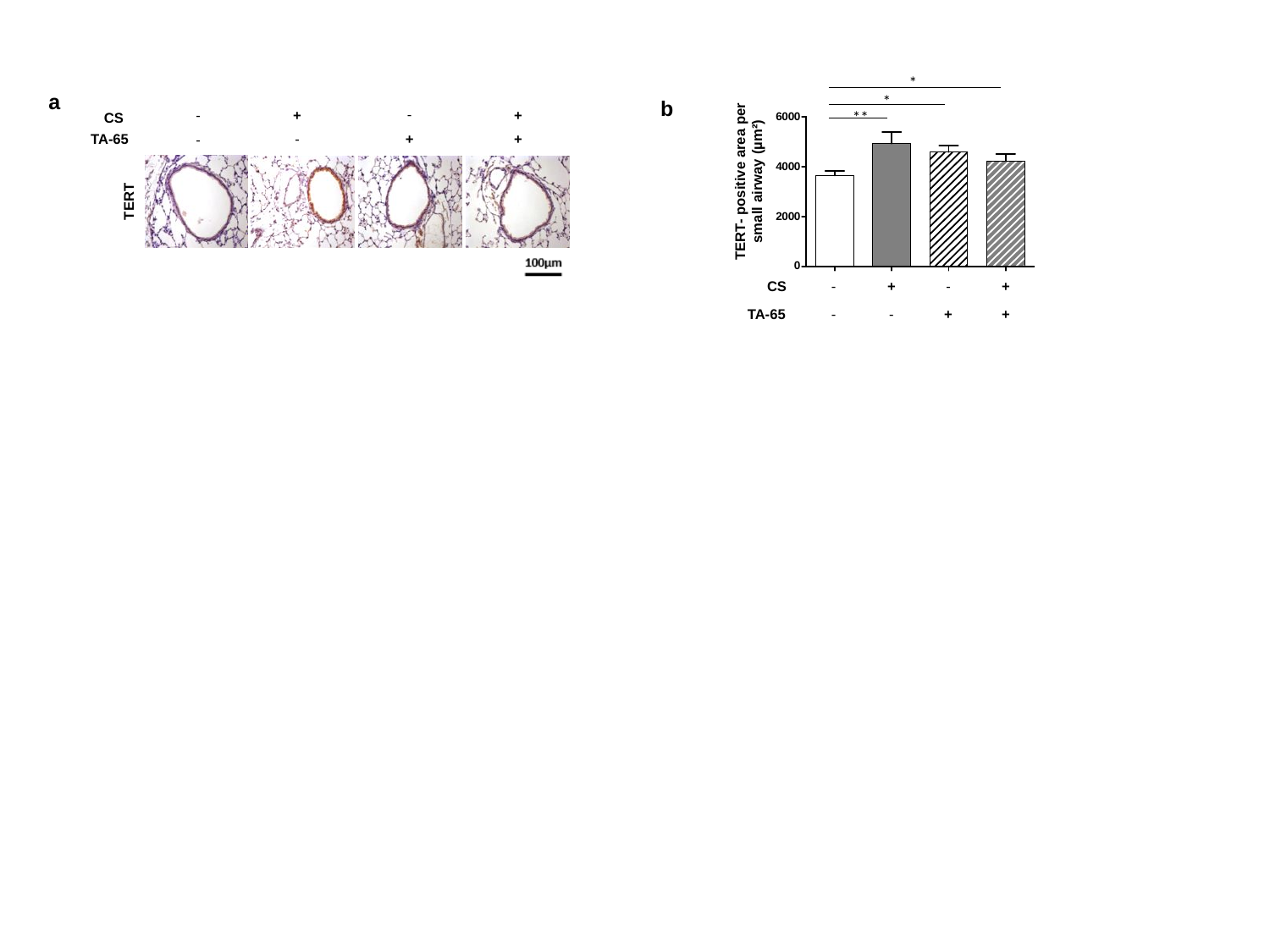

*
a
*
TERT- positive area per small airway (µm²)
b
-
-
**
+
+
CS
TA-65
-
+
+
-
TERT
CS
-
+
-
+
TA-65
-
-
+
+

Supplement: Supplementary file 1 — Supplementary Figure S1. [file 41598_2022_25993_MOESM1_ESM.pptx]

## Slide 1
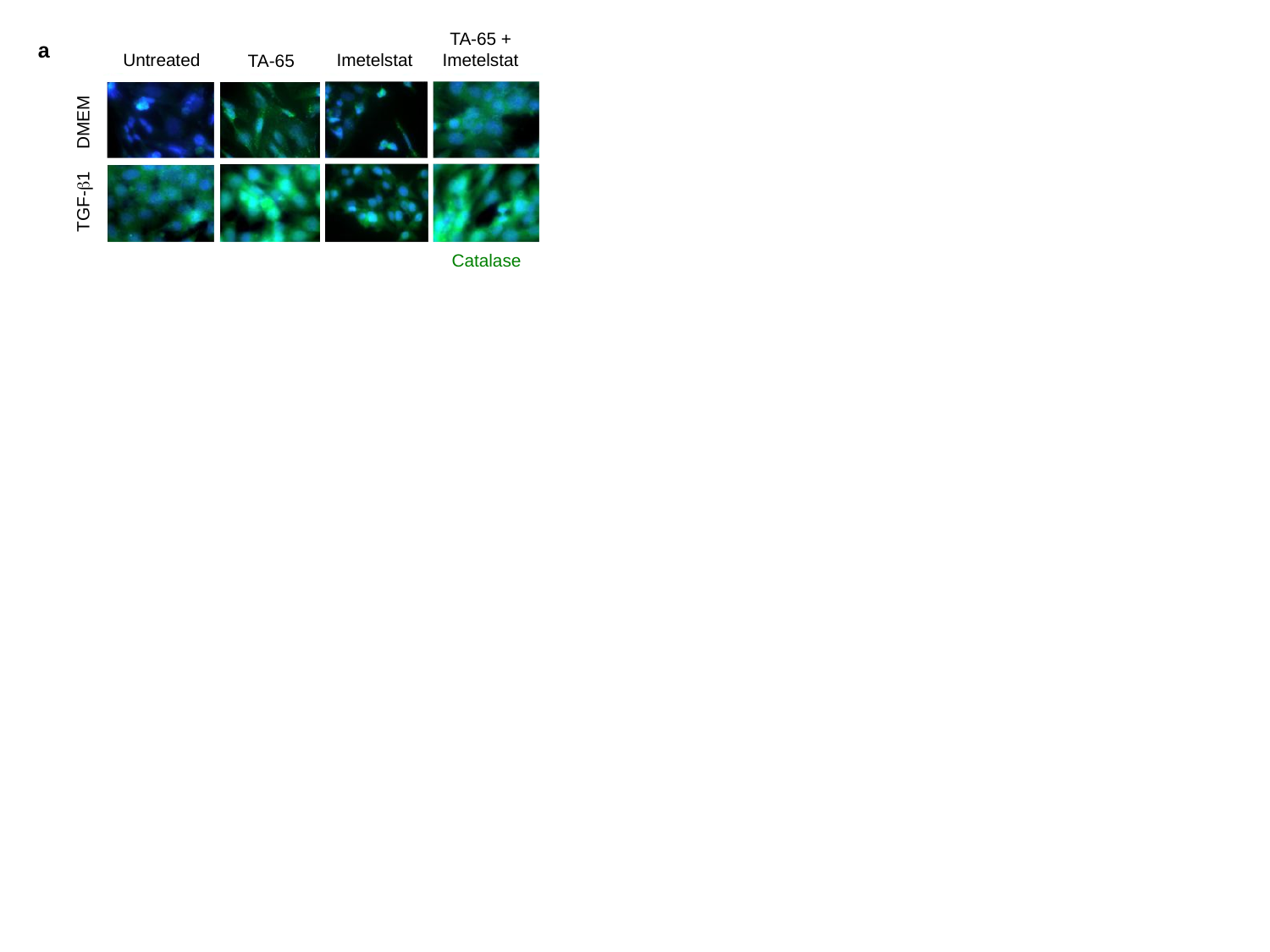

TA-65 + Imetelstat
Untreated
Imetelstat
TA-65
DMEM
TGF-b1
Catalase
a

Supplement: Supplementary file 4 — Supplementary Figure S4. [file 41598_2022_25993_MOESM4_ESM.pptx]
