# Supplementary material for: The telomerase activator TA-65 protects from cigarette smoke-induced small airway remodeling in mice through extra-telomeric effects
Source: Sci Rep. 2023 Jan 16;13:25. doi: 10.1038/s41598-022-25993-7 (PMC9842758; doi:10.1038/s41598-022-25993-7)
Supplement: Supplementary file 2 — Supplementary Figure S2. [file 41598_2022_25993_MOESM2_ESM.pptx]

## Slide 1
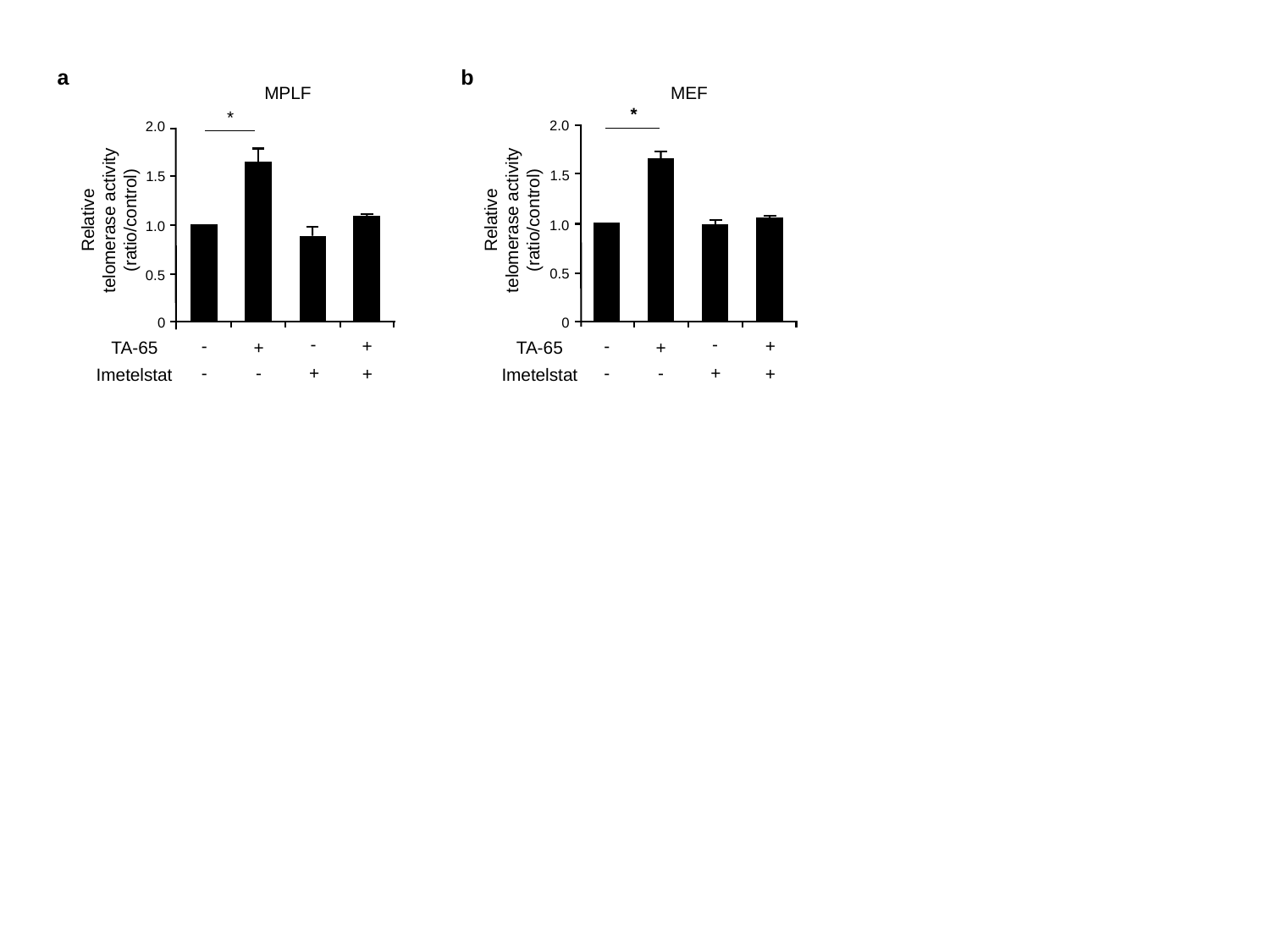

a
b
MPLF
MEF
*
*
2.0
2.0
Relative telomerase activity (ratio/control)
Relative telomerase activity (ratio/control)
1.5
1.5
1.0
1.0
0.5
0.5
0
0
-
-
+
TA-65
+
-
-
+
+
Imetelstat
-
-
+
TA-65
+
-
-
+
+
Imetelstat
