# Supplementary material for: The telomerase activator TA-65 protects from cigarette smoke-induced small airway remodeling in mice through extra-telomeric effects
Source: Sci Rep. 2023 Jan 16;13:25. doi: 10.1038/s41598-022-25993-7 (PMC9842758; doi:10.1038/s41598-022-25993-7)
Supplement: Supplementary file 3 — Supplementary Figure S3. [file 41598_2022_25993_MOESM3_ESM.pptx]

## Slide 1
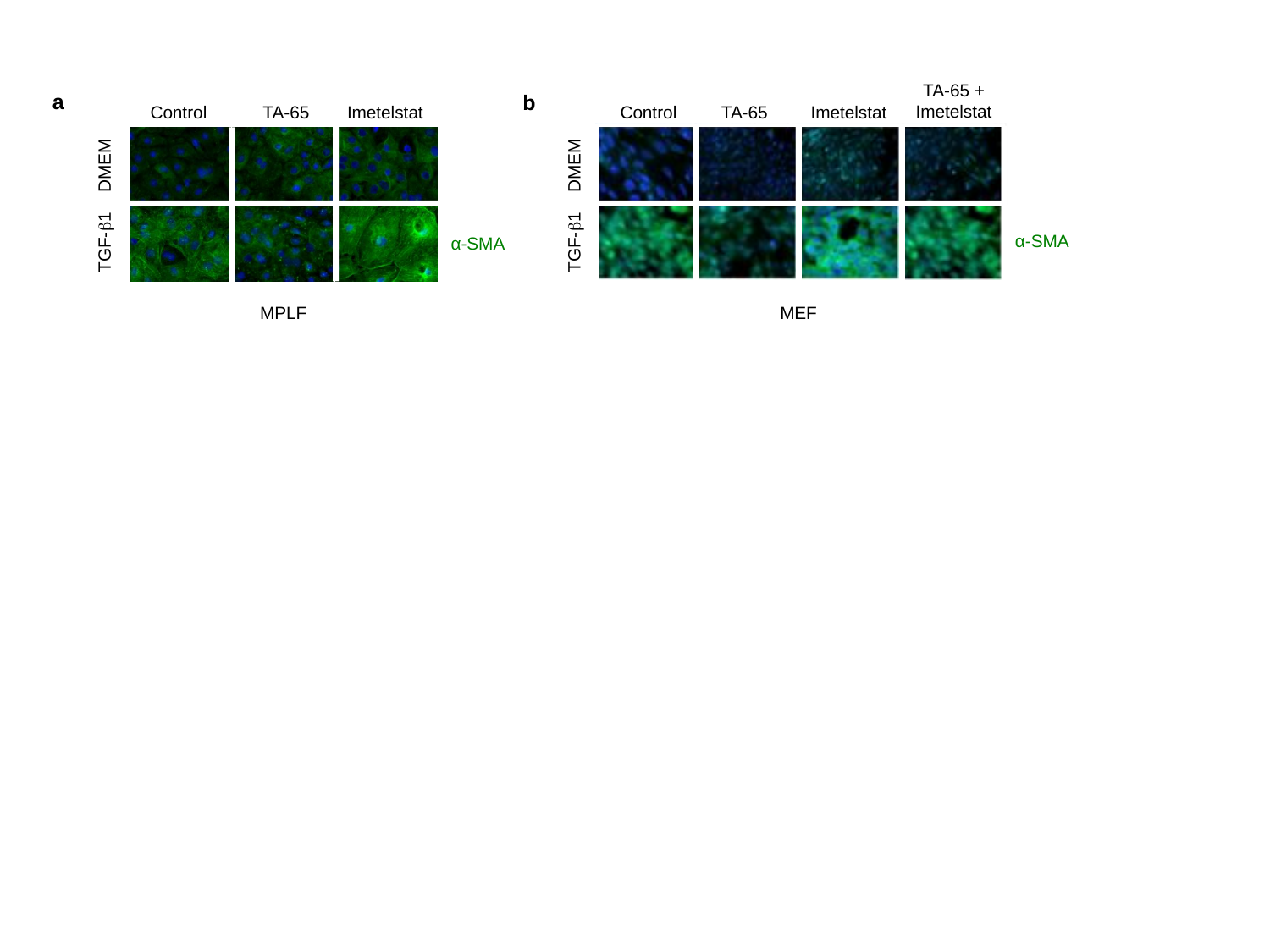

TA-65 + Imetelstat
a
b
TA-65
TA-65
Control
Imetelstat
Imetelstat
Control
DMEM
DMEM
TGF-b1
TGF-b1
α-SMA
α-SMA
MPLF
MEF
