# Supplementary material for: The telomerase activator TA-65 protects from cigarette smoke-induced small airway remodeling in mice through extra-telomeric effects
Source: Sci Rep. 2023 Jan 16;13:25. doi: 10.1038/s41598-022-25993-7 (PMC9842758; doi:10.1038/s41598-022-25993-7)
Supplement: Supplementary file 5 — Supplementary Legends. [file 41598_2022_25993_MOESM5_ESM.docx]

**Supplemental figures legends**

**Figure S1: TERT expression.** Representative lung tissue sections of mice exposed to air or cigarette-smoke (CS) ±TA-65, and subjected to TERT immunostaining (a). Quantification of TERT expression (b). *Original magnification, 20X. n = 7-8 mice per group. Data represent mean ± S.E.M. * p<0.05, **p<0.01.*

**Figure S2: Telomerase activity of lung fibroblasts *in vitro*.** Telomerase activity assessed in mouse primary lung fibroblasts (MPLF) treated with TA-65 (2 µM) of Imetelstat (1 µM) during 24 hours. *n = 3 samples per group. Data represent mean ± S.E.M. * p<0.05.*

**Figure S3:** **Effects of TA-65 on TGF-β-induced fibroblasts-to-myofibobroblasts differentiation *in vitro*.** Mouse primary lung fibroblasts (MPLF) and MEF cells were pre-treated with TA-65 (2 µM) or Imetelstat (1 µM) during 24 hours, followed by TGF-β treatment (10 ng/ml) for 48 hours: representative images of cells (a: MPLF; b: MEF) stained with α-SMA. Green stellate cells indicate the presence of myofibroblasts. *Original magnification, 40X.*

**Figure S4: Effects of TA-65, Imetelstat and TGF-β1 on catalase expression *in vitro*.** MEF cells were pre-treated with TA-65 (2 µM) and/or Imetelstat (1 µM) during 24 hours, followed by TGF-β treatment (10 ng/ml) for 48 hours. Catalase (in green) expression was evaluated by immunostaining (a). *Original magnification, 20X.*
